# Case of Injury of the Brain, with an Extensive Scalp Wound, and Fracture of the Skull

**Published:** 1859-01

**Authors:** W. T. Sherwood

**Affiliations:** East Paw Paw, Illinois


ARTICLE IV.

### CASE OF INJURY OF THE BRAIN, WITH AN EXTENSIVE SCALP WOUND, AND FRACTURE OF THE SKULL.

BY W. T. SHERWOOD, M. D., OF EAST PAW PAW, ILLINOIS.

*August* 27*th*, 1858.—Called at 8 o'clock a.m. to see Hiram Hampton, aged 14, who, an hour before, had been thrown from one of the horses attached to a reaper. The horses had become frightened, and were running as rapidly as the heft of the machine and other encumbrances would permit. One of the guards or teeth of the machine struck his head at or near the junction of the lambdoidal and squamous sutures of the left side, producing an extensive fracture of the parietal bone, and penetrating the brain to the depth of two or three inches. The fracture extended along the lambdoidal suture two and a half inches, and along or near the squamous suture three and one-fourth inches, and from this point upward toward the vertex about four inches, breaking this portion of the parietal bone into three pieces, leaving an opening in the skull, through which the brain protruded, of three or four inches square. A fracture of the occipital bone was also found of two or more inches in length, and extending downward and backward in a direct line with the sagittal suture. One of the pieces from the posterior inferior angle of the parietal bone presents a portion of the groove for the lateral sinus, which was ruptured and torn from its bed; the sulci, or grooves for the arterior meningia media, were also to be seen upon the bones that were removed, which, with the meninges, were torn from the brain. The pia mater, with several of the convolutions of the brain, were removed by the sickle guard, leaving the brain in a horribly mutilated condition. Many of the arteries of the scalp and brain were divided, and two or three inches of some of them entirely torn from the head. The posterior auricle, the temporal, the superior anterior branches of the occipital, and several of the large and smaller branches of the cerebral arteries were among the number injured. The amount of brain lost was estimated at half a gill. A piece of the scalp, measuring about two inches square, was nearly detached. The whole wound of the scalp measured over twelve inches in length. One incision extended from the vertex, near the junction of the sagittal, with the coronal suture obliquely downwards toward the ear, nearly five inches, and from this point posteriorly toward the occipital spine four or five inches more. Another extended from the posterior superior angle of the parietal bone, to a point just above the mastoid process of the temporal cutting, and turning up the scalp in two triangular pieces. One or two slight bruises were found upon the neck and shoulders and upon one of the hips. He was in an extreme state of prostration; pulse feeble and skin cold; he lies motionless and unconscious; if an effort is made to rouse him, he will answer questions, but reluctantly and with haste, and then relapse into a comatose insensible state again. When the wound was being dressed, he showed some signs of pain when the knife came in contact with the scalp. Locks of hair that had been severed from the head by the sickle were found driven one or two inches within the brain; these, with the pieces of fractured bone, were removed by the aid of the scalpel and forceps. The nearly detached portion of scalp, one or two of the convolutions of the brain that were badly lacerated, together with the portion of pia mater investing them, were also removed by the knife and scissors. A portion of the sound and uninjured scalp was then—as in operation for hare-lip—dissected from the skull and made to cover the unprotected portion of brain. Some twenty sutures were taken in the wound, bringing its edges closely together. Brandy and water was freely administered during the operation to keep him from sinking. At the close of the operation he appeared to be in an almost perfect state of collapse; pulse extremely feeble and intermittent; features pale and ghastly; surface cold; respiration slow, and performed very feebly. He was ordered to be wrapped in flannel, and warm applications made to the feet. Stimulants were administered at short intervals, until he partially recovered from this state of prostration. He remained in this condition until eight hours from the time he received the injury; he then, after vomiting several times, sank into a state of collapse again. He became profoundly insensible; his pulse ceased; his eyes became fixed, and his jaws set; his extremities and face were icy cold; trembling of the pectoral muscles, and one or two slight convulsions, were noticed. A dose of carbonate of ammonia, with a little brandy and water, brought on an imperfect state of reaction. His pulse came up to 80, but were very weak and irregular, often sinking to 40 beats per minute, and then again, after the administration of ammonia, rise to 70 or 80. When his pulse sank he appeared as if in a quiet tranquil sleep; his extremities would again become cold; his respiration slow, sighing and feeble; but when his pulse came up again he would be restless and feverish; his skin hot and dry, and his lips parched. His pulse remained in this changeable condition until the third day, when they became more regular,—seldom falling below 80 or rising above 90—and were full and soft. His appetite began to improve slightly; he appeared much more rational, but slept the greater part of the time. When aroused he seemed perfectly conscious of what was taking place about him; would answer when spoken to, get up and take food or drink when offered him, and would listen to conversation for an hour or more at a time. A mild cathartic was given for the first time on the eve of the third day. He had had two evacuations from the bowels since the accident occurred, but from the fact that it was not supposed possible that he could survive the injury more than thirty-six hours, and from the depletion that had already taken place from the loss of a great amount of blood, it was not thought advisable to deplete any farther, not even with cathartic. An enema was ordered to be given every two days if his bowels should become torpid. On the evening of the sixth day I found his pulse up to 96, but soft, very full and jerking; this was accompanied with an increased throbbing and heat of the brain. I ordered the cold cloths, which had been used from the time that reaction first came on, to be wet in very cold water, and changed as soon as they began to get warm, which was as often as every three minutes. A dose of magnesia sulph. was given, followed in three minutes with an enema. Veratrum vir. was left to be given every four hours in four drop doses. Called again on the evening of the next day: found him much more comfortable; pulse 76, and very soft. His appetite and strength seemed to increase from this time until the fourteenth day, his pulse remaining at 80. He was able to sit up in a chair when his bed was being made, and to converse with his parents for half an hour at a time. His memory was very much impaired, which he himself noticed and often spoke of. He remembered the occurrence of the accident, the horses running, and his being thrown off; he also knew and called people by name that called on him. A healthy scab formed over the wound, which, on the twelfth day, was thrown off, leaving the wound perfectly healed. Suppuration took place on the seventh day, and from that time until the fourteenth, the wound was doing as well as I could wish,—about eight inches of it being healed. I did not visit him on the thirteenth. Called on the fourteenth at 7 o'clock p.m. Found that inflammation of the brain had set in; his pulse 124; sleep disturbed; skin hot and dry; carotids pulsate violently; the tongue dry and the appetite gone; the stomach irritable and the bowels constipated; the pain in the head very severe; eyes intolerant to light; conjunctiva injected and red; impatience, irritability and constant agitation; arms continually raised towards the head, and is inclined to rest on the side injured. I left him veratrum again to be taken as before. Gave him an enema, which operated in about thirty minutes. Ordered the cold cloths to be changed frequently, and to be applied to the whole head. Called on the fifteenth day at 9 a.m. Found his pulse 96; irritability and restlessness much less; heat and dryness of the skin was also much diminished. On the eve of the same day, from suppuration and sloughing of the divided arteries, he lost some blood, which was checked with cold applications. The wound, which had before discharged a healthy laudable pus, now discharged a thin gleety matter, which ran down the neck in streams, wetting the pillows and bedding. On the sixteenth day he was again taken with bleeding from the divided arteries, which was not checked until syncope was produced. He remained in a semi-comatose state until the next morning (seventeenth day), when he quietly and without a struggle glided into the arms of death.

